# A Novel Neutrophil-Based Biomarker to Monitor Disease Activity and Predict Response to Infliximab Therapy in Patients With Ulcerative Colitis

**DOI:** 10.3389/fmed.2022.872831

**Published:** 2022-04-27

**Authors:** Zhou Zhou, Yinghui Zhang, Yan Pan, Xue Yang, Liangping Li, Caiping Gao, Chong He

**Affiliations:** ^1^Department of Gastroenterology, Sichuan Provincial People's Hospital, University of Electronic Science and Technology of China, Chengdu, China; ^2^Clinical Immunology Translational Medicine Key Laboratory of Sichuan Province, Sichuan Provincial People's Hospital, University of Electronic Science and Technology of China, Chengdu, China

**Keywords:** ulcerative colitis, biomarker, inflammatory bowel disease, neutrophil, albumin, neutrophil-to-albumin ratio, infliximab

## Abstract

**Background:**

Ulcerative colitis (UC) is characterized by refractory and recurrent mucosal inflammation, leading to a substantial healthcare burden. Diagnostic biomarkers predicting disease activity and treatment response remain elusive. To evaluate the application value of a novel neutrophil-based index (the neutrophil-to-albumin ratio, NAR) as a novel diagnostic biomarker in patients with UC and a predictive marker for disease activity and response to infliximab (IFX) therapy.

**Methods:**

Clinical characteristics and laboratory parameters of enrolled subjects (patients with UC and healthy controls) were retrieved from the electronic medical record database of our hospital. Serum cytokine and fecal calprotectin levels were measured by enzyme-linked immunosorbent assay (ELISA). Mucosal expression levels of inflammatory agents were measured by quantitative RT-PCR (qRT-PCR).

**Results:**

We found that NAR, which had not yet been explored in UC, was significantly increased in patients with UC (*n* = 146) compared to that in controls (*n* = 133) (1.95 ± 0.41 vs. 1.41 ± 0.23, *p* < 0.0001). NAR showed a positive association with the disease activity and inflammatory load in patients with UC. Pre-treatment NAR was significantly lower in IFX responders than that in non-responders (2.18 ± 0.29 vs. 2.44 ± 0.21, *p* = 0.0118), showing a significant ability to discriminate initial responders from primary non-responders to IFX induction therapy (AUC = 0.7866*, p* = 0.0076). Moreover, pre-treatment NAR predicted postinduction serum IFX trough level.

**Conclusion:**

Our study provides evidences to utilize NAR in the diagnosis, activity monitoring, and IFX response prediction in patients with UC.

## Introduction

Inflammatory bowel disease (IBD) is characterized by chronic and relapsing inflammation in the gastrointestinal tract, including two eminent forms, ulcerative colitis (UC) and Crohn's disease (CD). The prevalence of IBD is rapidly growing worldwide and rising health expenditures (1–4), leading to a substantial healthcare burden (5). In addition, IBD is medically refractory and has become a great clinical challenge.

Endoscopic biopsies have been recognized as the diagnostic gold standard in IBD. However, endoscopy has drawbacks that limit its application particularly in long-term follow-up, such as invasiveness, high cost, and inter-observer variability (6). In addition, patients may not tolerate endoscopic examinations very well and those who undergo endoscopy may complained embarrassment, discomfort caused by bowel preparation, and increased abdominal pain (7, 8).

Besides endoscopy, a panel of adjunctive serological biomarkers, which are much less-invasive, have been applied at nearly every point in the management of IBD. They have shown practical values to distinguish IBD from gastrointestinal functional disease, differentiate active from inactive status, and predict therapeutic effect, recurrence, prognosis (9).

Blood tests including erythrocyte sedimentation rate (ESR), C-reactive protein (CRP), fibrinogen, albumin, bilirubin, uric acid, auto-antibodies (e.g., anti-pTNP, ASCA antibodies) are widely utilized in IBD management (10–14), as well as fecal calprotectin and lactoferrin (15). Given the crucial role of neutrophils in the disease pathogenesis (16, 17), neutrophil-based indexes have been generated and applied in the IBD area (18–23). For example, the neutrophil-to-lymphocyte ratio (NLR) is easily accessible from routine blood tests for both inpatients and outpatients, and it has been strongly suggested as a valuable predictive marker to distinguish patients with IBD from non-IBD controls and differentiate disease activity in IBD (24).

Recently, the neutrophil-to-albumin ratio (NAR) has emerged as a sensitive index which indicates systemic inflammation and has been used in inflammatory, vascular diseases, and cancers (25–27). Since serum biochemical tests are also routine procedures to evaluate the nutritional status and screen renal/hepatobiliary dysfunctions in patients with IBD, NAR can be readily obtained for nearly any patient under care for IBD. However, to our best knowledge, this index has not yet been utilized in UC.

Therefore, we questioned whether NAR could be used as a reliable, non-invasive, and cost-effective biomarkers, regarding disease diagnosis, activity monitoring, or drug response prediction. In this study, we evaluated the application value of NAR as a diagnostic biomarker in patients with UC and a predictive marker for disease activity and response to infliximab (IFX) induction therapy.

## Methods

### Participants

This prospective study was conducted in accordance with the Declaration of Helsinki and approved by the Institutional Review Board for Clinical Research of Sichuan Provincial People's Hospital (No.201685, 2020204).

Patients with UC were consecutively recruited from the Department of Gastroenterology of Sichuan Provincial People's Hospital between January 2017 and December 2021. We also consecutively recruited non-IBD subjects, who underwent routine physical examinations in our hospital during the study. Participants enrolled in the current study were well-informed and signed an informed consent before participation. Patients with UC and controls were gender- and age-matched. Clinical characteristics and laboratory parameters of enrolled subjects were retrieved from the electronic medical record database of our hospital.

Exclusion criteria for both patients with UC and controls were as follows: smoking, excessive drinking, hematopoietic system disease, hepatobiliary disease, coagulation abnormalities, taking medications that can affect blood cell components or serum biochemistry profiles, hypertension, diabetes, infections, other systemic autoimmune diseases, other gastrointestinal diseases, and cancers. Demographics and clinical parameters of patients with UC (*n* = 146) and healthy controls (*n* = 133) were shown in Table 1.

**Table 1:** Demographics and clinical parameters of patients with UC and healthy controls.

	**UC**	**Healthy controls**	***p*-value**
Number of subjects (*n*)	146	133	-
Age (year)	38.5 ± 9.8	37.2 ± 10.4	0.2834*
Gender (*n*)			
Female	52	50	0.7319**
Male	94	83	
Disease duration (months)	35.4 ± 16.9	-	-
Disease extent (*n*)***			
E1	27	-	
E2	48	-	
E3	70	-	
Blood neutrophil (%)	69.66 ± 11.04↑	59.38 ± 8.52	<0.0001*
Serum albumin (g/L)	36.80 ± 7.31 ↓	42.40 ± 2.57	<0.0001*
CRP (mg/L)	31.29 ± 23.28	-	-
ESR (mm/hour)	53.84 ± 36.96	-	-
NAR	1.95 ± 0.41 ↑	1.41 ± 0.23	<0.0001*

*Data are presented as mean ± SD when applicable*.

*UC, ulcerative colitis; ESR, erythrocyte sedimentation rate; CRP, C-reactive protein; NAR, neutrophil-to-albumin ratio*.

*Student's t-test (unpaired, two-tailed), p < 0.05 was considered statistically significant.

**Chi-square test, p < 0.05 was considered statistically significant.

***Phenotypes of UC were classified according to the Montreal classification system.

*↑and↓: increased and decreased compared with healthy controls, respectively*.

As reported previously (28, 29), the diagnosis of UC was performed according to the comprehensive analysis of medical history, clinical manifestations, radiological, endoscopic, and histological examinations, as well as laboratory tests. The Mayo score system was utilized to determine the disease activity in patients with UC. To detailedly evaluate mucosal disease activity, the ulcerative colitis endoscopic index of severity (UCEIS) system was used. The disease extent of UC was identified based on the Montreal classification.

Infliximab (IFX) induction therapy was administered as reported previously (28). Briefly, patients with UC were infused with IFX (5 mg/kg) at weeks 0, 2 and 6 for induction. Short-term response to IFX were evaluated at 12–14 weeks post the initial infusion, when serum IFX trough levels were determined. Response (complete or partial) or primary non-response was defined by the physicians. To determine the predictive value of NAR in IFX response, patients were subjected to complete blood cell and serum biochemistry tests within 1 week before the first infusion of IFX, and pre-treatment NAR was calculated.

### Mucosal Inflammatory Agent Assessment

Mucosal biopsy tissues were collected during endoscopic examinations, immediately frozen in liquid nitrogen, and then sent to our laboratory for extended storage at −80°C. Total RNA was extracted from mucosal tissues using TRIzol reagent (Thermo Scientific). Reverse transcription for mRNA and microRNA (miR) were performed, respectively. Relative expression of cytokines and miR-301a was determined by qRT-PCR using a SYBR Green real-time PCR system (Invitrogen, CA, USA). The GAPDH and U6 expression levels were employed to normalize the expression of mRNA and miR, respectively (28).

### Measurement of Serum Cytokine and Fecal Calprotectin

Serum and fecal samples were collected, processed within 1 h, and stored at −80°C. Enzyme-linked immunosorbent assay (ELISA) was performed as described previously (28, 29). All ELISA kits (TNF-α, IFN-γ, calprotectin) were purchased from BioLegend (San Diego, CA, USA) and utilized according to the manufacturer's instructions.

### Statistical Analysis

Data are presented as mean ± SD when applicable. Chi-square test was performed to examine the difference of gender between patients with UC and controls. Comparisons between UC and controls regarding age and clinical parameters were performed by unpaired Student's *t*-test (two-tailed). The difference of NAR between IFX responders and primary non-responders was also examined by unpaired Student's *t-*test (two-tailed). The discriminating performance of a biomarker in indicated scenarios was determined by receiver operator curves (ROC) analysis. Correlations between two parameters were analyzed using Pearson's correlation analysis. *p* < 0.05 was set as statistically significant. All statistical analysis was performed using a Prism software Version 8.4 (Graphpad Software, San Diego, California, USA).

## Results

### Demographics and Clinical Parameters of the Participants

In the current study, 146 patients with UC (female = 52, male = 94) and 133 healthy controls (female = 50, male = 83) were included (Table 1). The mean age of patients with UC and healthy control was 38.5 ± 9.8 and 37.2 ± 10.4 years old, respectively. The duration of disease in patients with UC was 35.4 ± 16.9 months. There were no significant differences between patients with UC and healthy control regarding the age (*p* = 0.2834) and gender (*p* = 0.7319). Disease extent of UC was identified according to the Montreal classification system.

Neutrophil percentages were determined by complete blood cell tests, and albumin levels were examined by serum biochemistry tests. In line with existing evidences (11), patients with UC, compared to healthy controls, displayed a significant increase in neutrophil percentages (69.66 ± 11.04 vs. 59.38 ± 8.52 %, *p* < 0.0001) and a decrease in serum albumin levels (36.80 ± 7.31 vs. 42.40 ± 2.57 g/L, *p* < 0.0001).

Next, we calculated NAR as the ratio of neutrophil percentages over serum albumin levels and found that NAR was up-regulated by 1.4-fold in patients with UC compared with in healthy controls (1.95 ± 0.41 vs. 1.41 ± 0.23, *p* < 0.0001).

**Figure 1:**
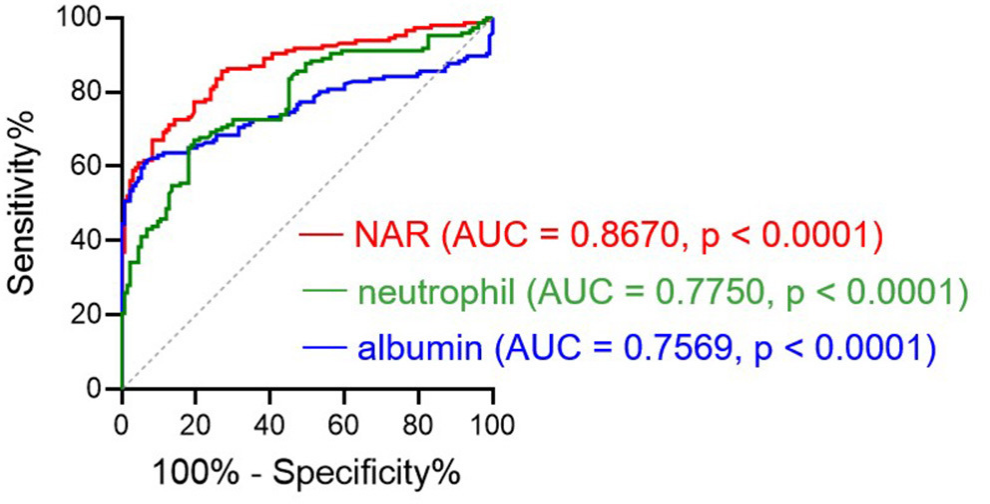
Receiver operating characteristics (ROC) curve analysis. The performances of the neutrophil-to-albumin ratio (NAR), blood neutrophil percentages, and serum albumin levels to discriminate ulcerative colitis (UC) from controls were determined by ROC curve analysis. AUC, area under the ROC curve. *p* < 0.05 was considered significant. UC, *n* = 146; Controls, *n* = 133.

In regard to the ability to discriminate UC from controls, we performed ROC analysis (Figure 1), showing that NAR had larger AUC (AUC = 0.8670) compared to neutrophil (AUC = 0.7750) or albumin (AUC = 0.7569) alone. Taken together, these data suggest that NAR could be a practical, rapid, and easily accessible biomarker for UC diagnosis.

### NAR Reflects Disease Activity in UC

**Figure 2:**
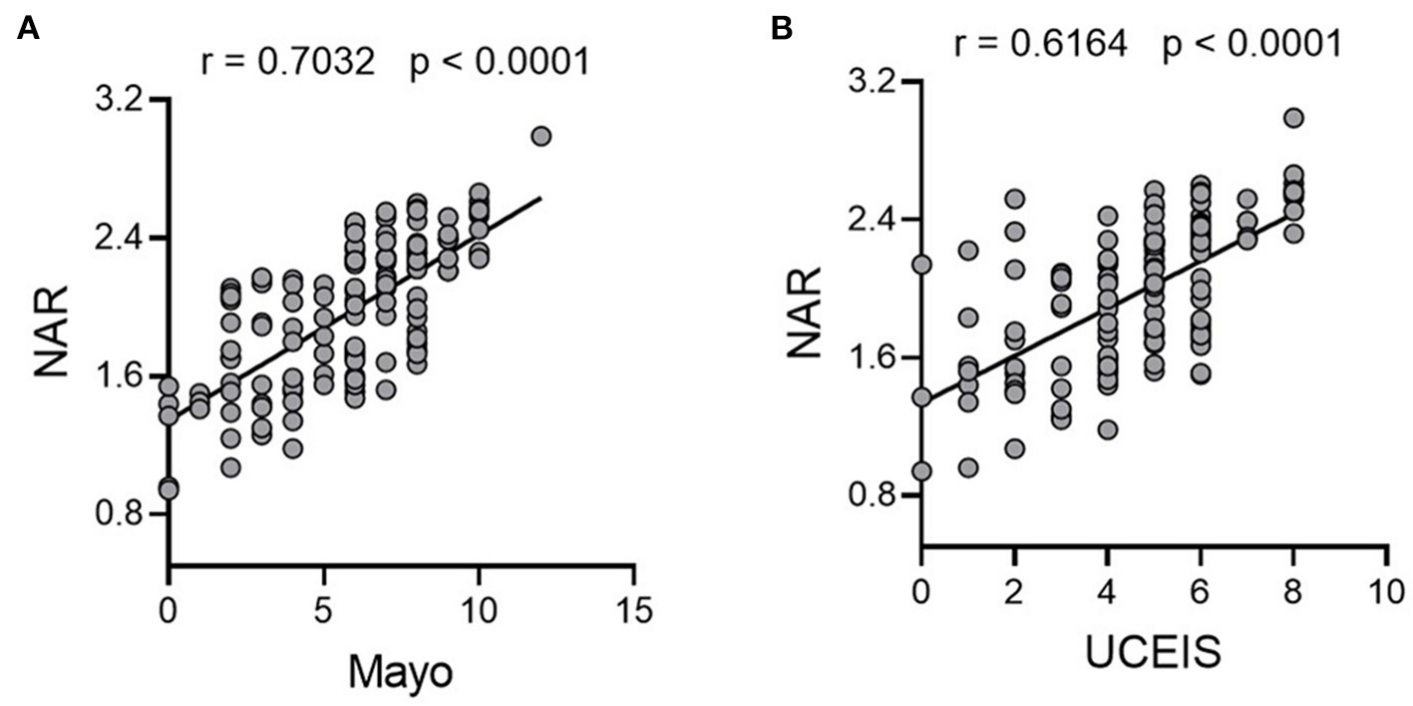
NAR reflects disease activity in UC. All enrolled patients with UC (*n* = 146) were subjected to disease activity evaluation by the Mayo score and the ulcerative colitis endoscopic index of severity (UCEIS) system. Associations between NAR and **(A)** Mayo score, and **(B)** UCEIS were examined. The correlation analysis was performed using Pearson's correlation. *p* < 0.05 was considered significant.

Since NAR could be used as a diagnostic biomarker for UC, we moved forward to explore whether this marker was able to distinguish UC activity. We looked at the potential association between NAR and Mayo scores, and patients with higher NAR exhibited more severe disease activity (Figure 2A, Pearson r = 0.7032, *p* < 0.0001).

Additionally, mucosal healing has been highlighted in the management of IBD and amounts of evidences have stressed that patients with mucosal healing display a lower rate of hospitalization and better prognosis (30–33). Therefore, we also employed UCEIS, which had a high interobserver agreement rate (34, 35), as an additional system to detailedly investigate the correlation between NAR and the endoscopic activity of patients with UC. As shown in Figure 2B, NAR showed a positive association with mucosal disease activity (Pearson r = 0.6164, *p* < 0.0001).

### Associations Between NAR and Inflammatory Load in Patients With UC

**Figure 3:**
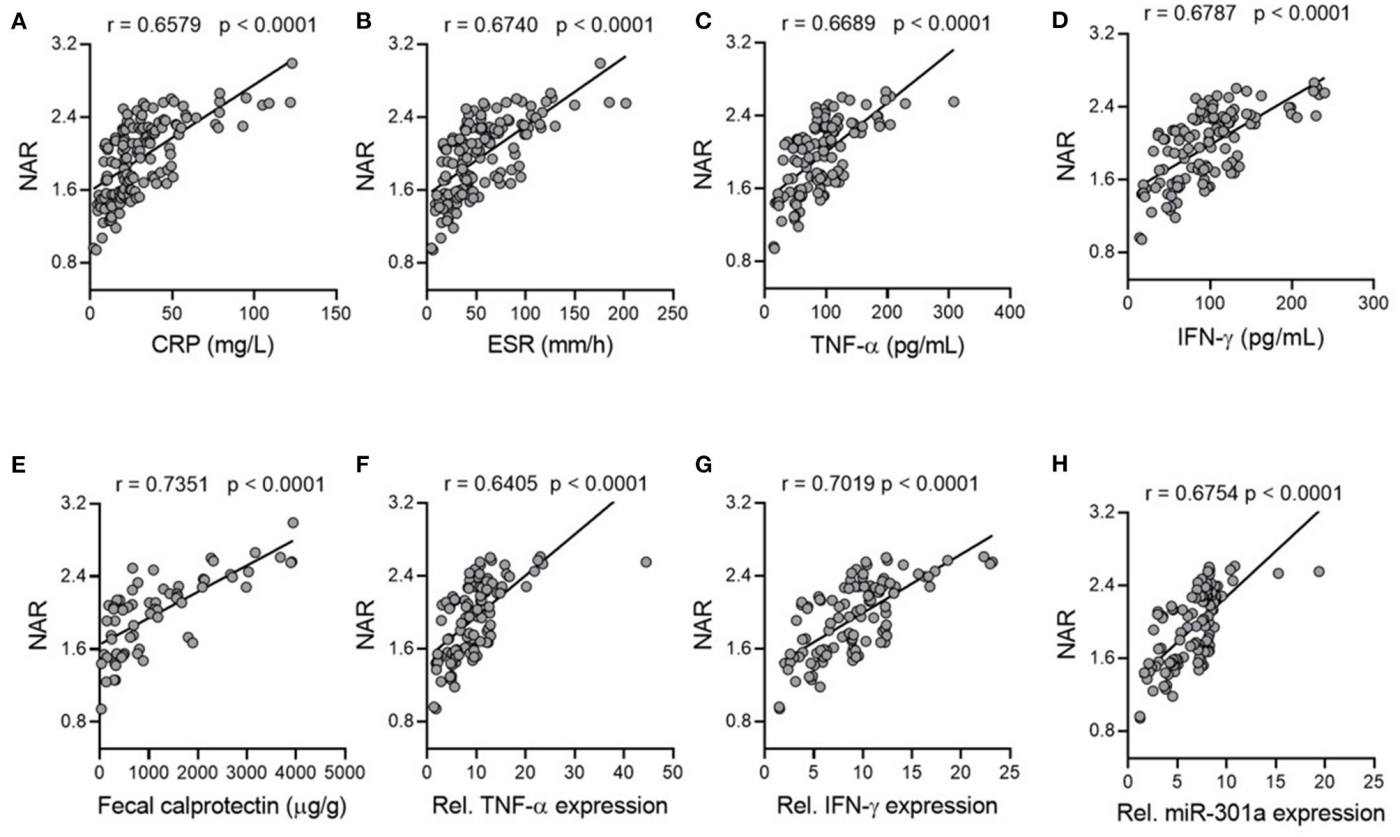
Associations of NAR with inflammatory load in patients with UC. Correlation of NAR with **(A)** C-reactive protein (CRP) and **(B)** erythrocyte sedimentation rate (ESR). **(A,B)**, *n* = 146. Serum **(C)** TNF-α and **(D)** IFN-γ protein levels were positively associated with NAR. **(C,D)**, *n* = 122. **(E)** Fecal calprotectin levels (*n* = 66), mucosal **(F)** TNF-α, **(G)** IFN-γ, and **(H)** miR-301a relative expression levels positively correlated with NAR. **(F–H)**, UC, *n* = 110; Controls, *n* = 75 (for gene relative expression calculation).

Both mucosal and systemic inflammatory conditions are related to disease severity in patients with UC, and higher inflammatory responses tend to indicate more severe disease activity and poorer drug responses (36). To this end, we first looked at serum inflammatory factors. CRP and ESR are the most commonly used disease activity biomarkers of IBD and up-regulated serum pro-inflammatory cytokines such as TNF-α and IFN-γ are hallmarks of IBD. We found that NAR was positively correlated with CRP, ESR, serum TNF-α and IFN-γ (Figures 3A–D), suggesting that NAR can be utilized as a marker reflecting the systemic inflammatory load in patients with UC.

Subsequently, we examined associations between NAR and mucosal inflammatory responses. Since neutrophilic accumulation in the inflamed mucosa is intimately associated with the disease activity in UC, it has been suggested that detection of inflammatory proteins secreted by neutrophils in the feces may be a promising procedure for mucosal inflammation assessment (37). Fecal calprotectin is a neutrophil-derived protein that has been applied as a reliable screening tool for intestinal inflammation due to its sensitivity. As shown in Figure 3E, NAR correlated well with fecal calprotectin levels in UC.

Next, we analyzed mucosal expression of TNF- α and IFN- γ mRNA, both of which were also positively associated with NAR (Figures 3F,G). In addition, our previous studies have demonstrated that mucosal miR-301a is a useful biomarker for diagnosis and disease activity in UC (28, 29). Therefore, we employed mucosal expression of miR-301a as an inflammatory factor of patients with UC, which was positively associated with NAR (Figure 3H). Collectively, our findings demonstrate that NAR, which is more convenient and faster than cytokine and fecal protein measurement, might assist to evaluate the systemic and mucosal inflammatory load in patients with UC.

### NAR Predicts Response to IFX in Patients With UC

The therapy for IBD has been revolutionized, thanks to biologics, especially anti-TNF agents (such as IFX), which are effective to induce and maintain remission in patients with both UC and CD. However, IFX does not work well in each patient, and about 65% of patients with UC respond to IFX induction therapy and the rate drops down to 45.5% during the maintenance therapy (38). Therefore, efforts have been made to identify reliable predictive biomarkers for both short- and long-term response to IFX.

**Figure 4:**
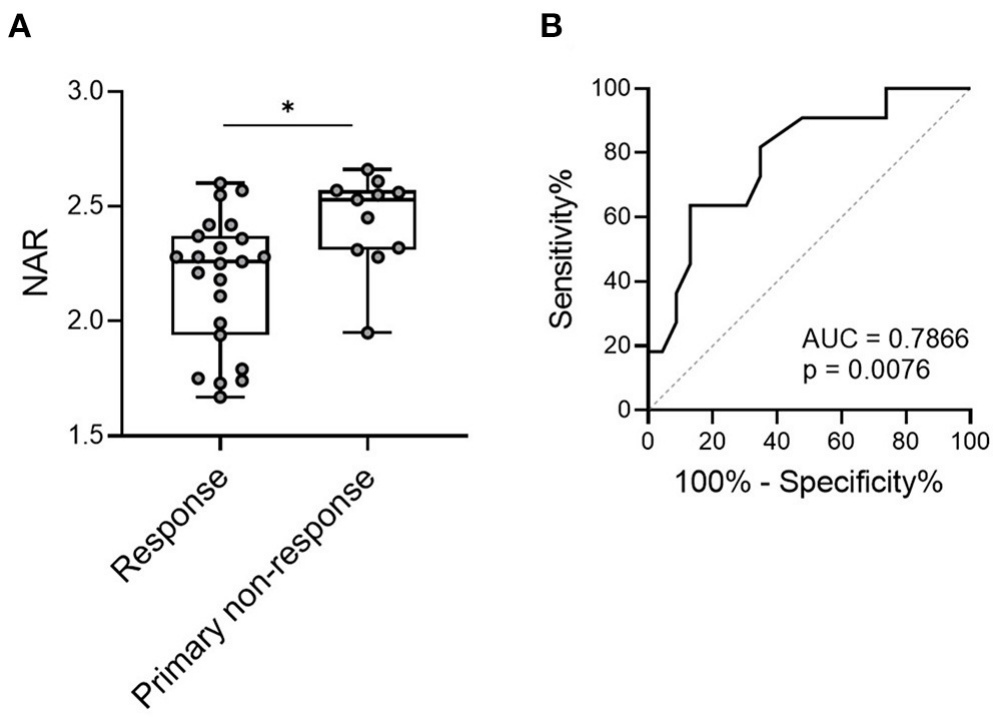
NAR predicts short-term response to infliximab (IFX) in patients with UC. Patients with UC (*n* = 34) were infused with IFX (5 mg/kg) at weeks 0, 2 and 6 for induction. Short-term response to IFX were evaluated at 12–14 weeks post initial infusion. Patients were subjected to complete blood cell and serum biochemistry tests within 1 week before the first infusion of IFX, and pre-treatment NAR was calculated. **(A)** Comparison of pre-treatment NAR was performed between responders (complete and partial, *n* = 23) and primary non-responders (*n* = 11). *p < 0.05, unpaired Student's *t*-test (two-tailed). **(B)** The ability of NAR to discriminate IFX responders from primary non-responders were determined by ROC curve analysis. *p* < 0.05 was considered significant.

To this end, we questioned whether NAR played as a predictor in this scenario. We enrolled 34 patients with UC, who were treated with IFX therapy (5 mg/kg at weeks 0, 2 and 6), including 23 responders (both complete or partial) and 11 with primary non-responders (evaluated at 12–14 weeks after the initial infusion). Patients were subjected to complete blood cell and serum biochemistry tests within 1 week before the first infusion of IFX, and pre-treatment NAR was calculated. As shown in Figure 4A, pre-treatment NAR was significantly lower in IFX responders than that in non-responders (2.18 ± 0.29 vs. 2.44 ± 0.21, *p* = 0.0118).

Next, we performed the receiver operating characteristics (ROC) curve analysis to determine the discriminative performance of NAR as predictors for response to IFX induction. NAR exhibited a significant ability to discriminate between initial responders and primary non-responders to IFX induction therapy (Figure 4B, AUC = 0.7866, *p* = 0.0076).

### NAR Predicts Serum IFX Trough Level (SIFX TL) in Patients With UC

**Figure 5:**
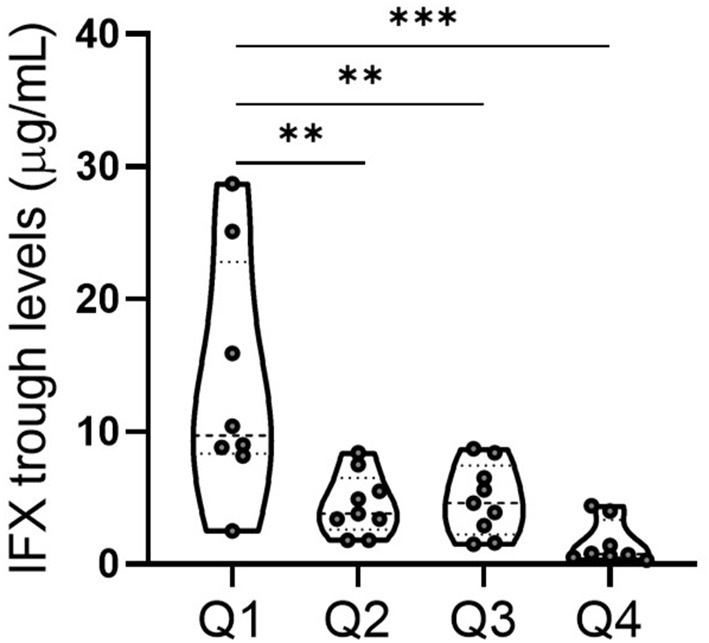
Association between NAR and serum IFX trough level (sIFX TL) in patients with UC. All 34 patients who received IFX induction therapy were grouped by quartiles of NAR. Q1 (quartiles 1, *n* = 8): 1.67 ≤ NAR < 2.11; Q2 (quartiles 2, *n* = 9): 2.11 ≤ NAR < 2.31; Q3 (quartiles 3, *n* = 9): 2.31 ≤ NAR < 2.55; Q4 (quartiles 4, *n* = 8): 2.55 ≤ NAR. sIFX TL in each group were measured. **p < 0.01, ***p < 0.001. One-way ANOVA followed by Tukey's multiple comparisons test was performed and adjusted *p*-values were calculated.

Since one contributing factor of primary non-response is low serum trough levels in IFX-treated patients (39, 40), and IFX clearance is accelerated in patients with high inflammatory load, causing insufficient clinical response (41, 42). As presented above, we demonstrated that NAR was a reflective marker of systemic and mucosal inflammatory conditions and higher NAR indicated lower rate of IFX response. Therefore, we sought to explore whether NAR was associated with postinduction sIFX TL. As shown in Figure 5, all 34 patients who received IFX therapy were grouped by quartiles of NAR and patients in quartiles 1 (≥ 1.67– < 2.11) exhibited significantly higher sIFX TL than those in other 3 quartiles. The lowest sIFX TL was observed in quartiles 4 (≥ 2.55), and no differences was found between quartiles 2 (≥ 2.11– < 2.31) and 3 (≥ 2.31– < 2.55). Collectively, these observations suggest that pre-treatment NAR might be a potential predictor for postinduction sIFX TL.

## Discussion

In the current study, we analyzed the alterations of a novel neutrophil- and albumin-based biomarker in patients with UC, demonstrating that: (1) NAR was significantly up-regulated in patients with UC compared with that in controls; (2) NAR showed significantly positive associations with the disease activity and inflammatory load in UC; (3) higher pre-treatment NAR indicated lower rate of short-term response to IFX therapy in patients with UC; (4) pre-treatment NAR predicted postinduction sIFX TL. These observations prompt us to speculate that NAR may be a promising biomarker in the diagnosis, activity monitoring, and IFX response prediction in patients with UC.

Although endoscopic examinations are the most powerful tool that can directly identify the lesion extent, location, and inflammation activity, several limitations are noteworthy such as high cost, inconvenience, and invasiveness, making this procedure unsuitable for frequent, long-term surveillance of UC. Given the advantage of non-invasiveness, serological and fecal biomarkers that can accurately reflect the gastrointestinal mucosal inflammatory status have become a resurgent interest in the IBD area (10).

To date, a number of such biomarkers have been reported (6, 43). As the most commonly used biomarkers, both CPR and ESR are rapid, easily-measured, and sensitive in the assessment of IBD activity. However, they are not disease specific and rise fast under conditions other than IBD (tissue necrosis, infection, or other causes), which largely limits their practical significance (15).

Fecal calprotectin is now one of the most sensitive biological indicators for discriminating IBD from bowel dysfunction, and the endoscopically active from the inactive (37). However, for some patients, stool-based tests might not be their first choice, probably related to a reluctance to collect and process fecal samples or high cost. Therefore, rapid, convenient, inexpensive, standardized, and reproducible blood-based tests may be a preferable option for most patients who need long-term monitoring.

Complete blood cell and serum biochemical tests are two most common routine examinations for the management of both inpatients and out patients with IBD. In the current study, we employed a newly-identified index calculated as the ratio of neutrophil percentages (from complete blood cell tests) over serum albumin levels (from serum biochemical tests). Since excessive mucosal neutrophil infiltration is a remarkable feature of UC, which plays a crucial role in crypt abscesses and cryptitis, patients with mucosal histologic normalization display better clinical outcomes (44).

On the other hand, patients with IBD usually present nutritional deficiency, which leads to hypoalbuminemia. Moreover, albumin has been reported to have powerful antioxidant properties and could serve as serum biomarkers in oxidative stress/inflammation-associated disorders (45). It has been reported that patients with IBD have lower levels of serum albumin, and hypoproteinemia might reduce therapeutic efficiencies of biologics (46). In the current study, we identified a novel neutrophil- and albumin-based index NAR was significantly increased in patients with UC compared to that in healthy controls. More notably, NAR served as a reliable marker to reflect endoscopic activity and mucosal inflammatory responses in patients with UC.

Anti-tumor necrosis factor (TNF) therapies have revolutionized the treatment of patients with IBD. Particularly, IFX, as the most-investigated anti-TNF agent, is effective for induction and maintenance of clinical remission and mucosal healing in patients with moderate-to-severe UC, who have lower hospitalization and colectomy rates during follow-up compared to placebo-controls (38). However, about a third of IFX-treated patients with IBD fail to show significant clinical benefit after the induction therapy (14 weeks after the initiation infusion), which is called “primary non-response” (40). Furthermore, secondary non-response during the first year of maintenance therapy can be found in ~40% of patients (47).

Since IFX does not work in all patients and has possibilities of major adverse effects, as well as high expense, it is important to develop predictive biomarkers for the loss of response in patients. Progress has been made in this research field and a panel of biomarkers have been identified, such as patient-related (age, weight, smoke, et al.) and disease-related (disease duration, CRP, albumin, et al.) (46). Regrettably, most markers have not shown practical application and many others remain controversial (46). Here, we found that NAR, which integrated neutrophils and albumin, showed significant abilities to discriminate IFX responders from primary non-responders, providing a new approach for clinical practice.

A number of evidences have suggested that the loss of response to IFX correlates with low levels of serum IFX trough levels (41). In the current study, we observed that patients with higher pre-treatment NAR had lower serum IFX trough concentration. It has been reported that patients in high inflammatory status may have a higher rate of IFX clearance, leading to both insufficient clinical response and development of antibodies toward IFX (ATI) (42, 48, 49). We here demonstrated a role of NAR as an indicator for both systemic and mucosal inflammatory load and patients with more severe UC displayed higher NAR, which might promote IFX clearance and explain their loss of response to this agent.

In summary, we found a novel neutrophil- and albumin-based index NAR was significantly elevated in patients with UC, and showed satisfactory performance in the assessment of disease activity and inflammatory load. In note, NAR displayed predictive values of primary response to IFX induction therapy in patients with UC. Despite these findings, several issues need to be addressed in the following studies: studies with large sample sizes are needed to verify our current observations; whether NAR could be used in long-term follow-up to predict secondary non-response to IFX; the practical significance of NAR for other biologics (such as Vedolizumab and Ustekinumab) need to be investigated. Nevertheless, the current study provides evidences to utilize NAR in the diagnosis, activity monitoring, and IFX response prediction in patients with UC.

## Data Availability Statement

The original contributions presented in the study are included in the article/supplementary material, further inquiries can be directed to the corresponding authors.

## Ethics Statement

The studies involving human participants were reviewed and approved by the Institutional Review Board for Clinical Research of Sichuan Provincial People's Hospital (No.201685, 2020204). The patients/participants provided their written informed consent to participate in this study.

## Author Contributions

CH and CG conceptualized, designed the study plan, and edited the manuscript. ZZ, YZ, and XY collected clinical information and samples from enrolled subjects. LL, YP, and CG diagnosed the patients. CH, CG, and YZ analyzed the data and prepare the original draft. ZZ and CH revised the manuscript. All authors discussed, revised the manuscript, and agreed to the published version of the manuscript.

## Funding

This work was financially supported by grants from the National Natural Science Foundation of China (82070985 and 82170579) and Foundation of Sichuan Science and Technology Department (2021JDJQ0044).

## Conflict of Interest

The authors declare that the research was conducted in the absence of any commercial or financial relationships that could be construed as a potential conflict of interest.

## Publisher's Note

All claims expressed in this article are solely those of the authors and do not necessarily represent those of their affiliated organizations, or those of the publisher, the editors and the reviewers. Any product that may be evaluated in this article, or claim that may be made by its manufacturer, is not guaranteed or endorsed by the publisher.

## Abbreviations

IBD: inflammatory bowel disease
CD: Crohn's disease
UC: ulcerative colitis
NAR: neutrophil-to-albumin ratio
NLR: neutrophil-to-lymphocyte ratio
ROC: receiver operating characteristics
PCR: polymerase chain reaction
qRT-PCR: quantitative Real-time PCR
ELISA: enzyme-linked immunosorbent assay
IFX: infliximab
ESR: erythrocyte sedimentation rate
CRP: C-reactive protein
TNF: tumor necrosis factor
IFN: interferon
miR: microRNA
UCEIS: ulcerative colitis endoscopic index of severity
AUC: area under the curve.
